# Improved VO_2max_ Estimation by Combining a Multiple Regression Model and Linear Extrapolation Method

**DOI:** 10.3390/jcdd10010009

**Published:** 2022-12-27

**Authors:** Tomoaki Matsuo, Rina So, Fumiko Murai

**Affiliations:** 1 Ergonomics Research Group, National Institute of Occupational Safety and Health, Japan, Kawasaki 214-8585, Japan; 2 Research Center for Overwork-Related Disorders, National Institute of Occupational Safety and Health, Japan, Kawasaki 214-8585, Japan

**Keywords:** cardiorespiratory fitness, exercise test, occupational health, physical fitness

## Abstract

Maximal oxygen consumption (VO_2max_) is an important health indicator that is often estimated using a multiple regression model (MRM) or linear extrapolation method (LEM) with the heart rate (HR) during a step test. Nonetheless, both methods have inherent problems. This study investigated a VO_2max_ estimation method that mitigates the weaknesses of these two methods. A total of 128 adults completed anthropometric measurements, a physical activity questionnaire, a step test with HR measurements, and a VO_2max_ treadmill test. The MRM included step-test HR, age, sex, body mass index, and questionnaire scores, whereas the LEM included step-test HR, predetermined constant VO_2_ values, and age-predicted maximal HR. Systematic differences between estimated and measured VO_2max_ values were detected using Bland–Altman plots. The standard errors of the estimates of the MRM and LEM were 4.15 and 5.08 mL·kg^−1^·min^−1^, respectively. The range of 95% limits of agreement for the LEM was wider than that for the MRM. Fixed biases were not significant for both methods, and a significant proportional bias was observed only in the MRM. MRM bias was eliminated using the LEM application when the MRM-estimated VO_2max_ was ≥45 mL·kg^−1^·min^−1^. In conclusion, substantial proportional bias in the MRM may be mitigated using the LEM within a limited range.

## 1. Introduction

Cardiorespiratory fitness (CRF) strongly influences disease incidence and is therefore an important health indicator [1,2,3]. Nevertheless, CRF is not routinely assessed in clinical practice [4], primarily because of assessment challenges. The measurement of maximal oxygen consumption (VO_2max_), the gold standard metric for CRF evaluation, is time-intensive and requires skilled examiners as well as specialized equipment (e.g., a treadmill/cycling ergometer and a gas analyzer). A field-based exercise test, such as the 20 m shuttle run [5], is an alternative for facilitating efficient mass screening of CRF. This validated shuttle run test is convenient for children because it can be performed in conjunction with a sporting event or physical education class in the school gymnasium or sports field; in contrast, it is much less convenient for adults because such facilities are rarely available in the workplace.

A questionnaire-based, non-exercise VO_2max_ estimation [6,7,8,9] has been developed for population-based CRF assessments. However, such non-exercise methods have been shown by some previous studies [10,11] to have insufficient accuracy, suggesting that biological data such as the heart rate (HR) should be included in VO_2max_ estimation because only the participants’ subjective responses may not correspond with VO_2max_ variance. Therefore, the use of HR in simple exercise tests such as the step test has the potential to be applied to CRF evaluation in adults, as it can be performed without skilled examiners, expensive equipment, a large exercise space, and participants’ maximal exercise effort.

A multiple regression model (MRM) is often used in the development of estimated VO_2max_ (eVO_2max_) equations, with measured VO_2max_ (mVO_2max_) as the response variable and clinicodemographic variables and/or other measurement variables, such as HR and physical activity (PA) parameters, as the explanatory variables [6,7,8,9,12,13,14]. Two studies [15,16] showed that the addition of HR during a step test to a questionnaire-based, non-exercise MRM improved the accuracy of VO_2max_ estimation. On the other hand, the Chester step test [17], which is considered an appropriate evaluation tool for CRF in adults owing to its acceptable reliability [18,19], uses the linear extrapolation method (LEM). The LEM is based on the use of HR along with predetermined VO_2_ values as constants during a stepping exercise. Subsequently, the best-fit line for HR and VO_2_ is drawn, and the line is extrapolated up to eVO_2max_, which corresponds to the age-predicted maximal HR. 

The MRM and LEM play an important role in VO_2max_ estimation; however, each of these two methods has its own inherent problems. In particular, the MRM-predicted values are distorted at regression edges, creating systematic errors [20]. Typically, the MRM either underestimates the VO_2max_ in participants with high CRF or overestimates the VO_2max_ in those with low CRF [7,8,9,14]. On the other hand, the LEM usually does not introduce systematic errors but yields a larger error variance than the MRM [14], which is likely due to the inaccuracy of the predicted maximal HR [21,22]. A previous study [16] reported that the addition of HR data from a step test to a questionnaire-based, non-exercise MRM could improve the estimation power of eVO_2max_ in cross-sectional analyses. However, VO_2max_ estimation using the developed MRM was problematic in detecting increased VO_2max_ induced by lifestyle changes because the predicted values were distorted at the upper limit, resulting in a VO_2max_ underestimation among participants whose actual VO_2max_ was improved by their exercise lifestyle. Additionally, the study showed that the apparent systematic errors (underestimation of high values) seen in the MRM were absent in the LEM, that the LEM could detect the increased VO_2max_ induced by the exercise intervention, and that the estimated error of the LEM was generally greater than that of the MRM.

Therefore, a comparison of accuracy between VO_2max_ estimation using the MRM and VO_2max_ estimation using the LEM on data from the same participants may facilitate the construction of a combined model with the greatest possible accuracy. We speculated that the most accurate estimate of VO_2max_ could be achieved through the use of a combined model in which the LEM corrected for VO_2max_ underestimation by the MRM in participants with a high level of fitness.

In the present study, we applied the eVO_2max_ MRM equation, including questionnaire scores and step-test HRs, which was developed in a previous study [16], to participants who were different from those in the equation development study and investigated the cross-validity of this equation. Subsequently, using this dataset, we aimed to investigate an accurate VO_2max_ estimation method that combines the advantages of both MRM and LEM and mitigates the weaknesses of these two methods.

## 2. Materials and Methods

### 2.1. Participants

Adults aged 30–60 years who were residents of the Tokyo area, were working part-time or full-time at least three days per week, did not use any drugs that influence the autonomic nervous system (e.g., β-blockers), and had no medical conditions precluding VO_2max_ testing were included as study participants. The participants were recruited via website advertisements between June 2016 and March 2022. A total of 137 working adults (65 women and 72 men) participated in this study. The participants visited our laboratory on two occasions, with an interval of 1 week between these visits. On the first visit, the participants underwent anthropometric measurements and a step test and then responded to a questionnaire. On the second visit, the participants underwent a treadmill exercise test for actual VO_2max_ measurement. Nine participants were excluded because of insufficient data for analyses. Consequently, 128 participants (60 women and 68 men) were included in the analysis. This study was conducted in accordance with the principles embodied in the Declaration of Helsinki. The study protocol was reviewed and approved by the Ethics Committee of the National Institute of Occupational Safety and Health, Japan (approval ID: H2744; date of approval: 31 March 2016). Written informed consent was obtained from all participants after providing a full explanation of the study aims and research protocols.

### 2.2. Measures

#### 2.2.1. Anthropometric Measurements

Height was measured once to the nearest 0.1 cm, whereas body weight was measured once to the nearest 0.1 kg. The body mass index (BMI) was calculated as weight in kilograms divided by the square of height in meters.

#### 2.2.2. Questionnaire

The questionnaire used in this study was previously validated for VO_2max_ estimation [7]. It consists of several questions regarding the frequency, duration, and intensity of PA, yielding a total PA score of 0–44 points. A higher PA score suggested a higher VO_2max_. The questions and scores assigned to each are presented in Table S1.

#### 2.2.3. Step Test

The step test used in this study, named the National Institute of Occupational Safety and Health, Japan (J-NIOSH) step test (JST), was previously validated for VO_2max_ estimation [14]. It comprises three 1 min stepping exercise stages followed by two 1 min recovery stages. In the exercise stages, the participants were required to step up and down a 30 cm step in time with a metronome beating at four times the step rate. The initial step rate (stage 1) was 15 steps/min (60 beats per min [bpm]) and was increased by 5 steps/min (20 bpm) for each subsequent stage (to a final step rate of 25 steps/min or 100 bpm on the metronome during stage 3). At the end of stage 3, the participants rested in the sitting position, and HR recordings were obtained at 1 and 2 min during the recovery stage. The protocol for the JST allowed the participants to skip stage 3 if the following two criteria were met at the end of stage 2: (i) HR of 80% of the age-predicted maximal HR (i.e., 220—age in years) and (ii) a rating of perceived exertion (RPE) of 17 on the Borg scale. However, none of the participants skipped stage 3 in this study. The HR index was calculated as follows: *HR index* = (*HR at exercise stage 3* − *HR at exercise stage 1*) + (*HR at recovery stage 1* − *HR at recovery stage 2*). 

#### 2.2.4. mVO_2max_

In order to directly measure the VO_2max_, the participants performed an exhaustion-limited graded exercise test on a treadmill (AR200; Minato Medical Science, Osaka, Japan) using the Bruce protocol. During the test, the ventilation and expired gases were continuously measured using an open-circuit computerized indirect calorimeter (AE-310S; Minato Medical Science, Osaka, Japan) that was calibrated prior to each trial. The HR was monitored using an electrocardiogram (Life Scope, Nihon Kohden, Tokyo, Japan). The RPE was recorded using the Borg 6–20 scale. The highest 30 s average VO_2_ value was defined as the VO_2max_ value when three of the following four criteria were satisfied: (i) the respiratory exchange ratio exceeded 1.10; (ii) the maximal HR was within 10 bpm of the age-predicted maximum (i.e., 220—age in years); (iii) the RPE exceeded 17; and (iv) the VO_2_ reached a plateau despite further increases in workload [23,24]. 

#### 2.2.5. eVO_2max_

The MRM eVO_2max_ was calculated using the following equation derived from a previous study [16]: *eVO_2max_ (MRM) = 64.22 − (0.23 × age) + (5.74 × sex; women = 0 and men = 1) − (0.57 × BMI) + (0.19 × questionnaire’s PA score) − (0.18 × JST’s HR index).*

The LEM eVO_2max_ was calculated based on a previous study [16]. Briefly, the LEM used a predetermined constant VO_2_ value for each stage of the step test. The constant values were 4, 13, 19, 22, 17, and 8 mL·kg^−1^·min^−1^ for females and 4, 14, 20, 23, 18, and 8 mL·kg^−1^·min^−1^ for males at rest, during exercise stages 1–3, and during recovery stages 1 and 2, respectively. A scatter plot was constructed for each participant, with six predetermined constant VO_2_ values (mL) for each stage of the JST (at rest, during exercise stages 1–3, and during recovery stages 1 and 2) on the *x*-axis and HR (bpm) on the *y*-axis. The best-fit line was calculated for the scatter plot, and the VO_2_ value (*x*-axis) corresponding to the participant’s age-predicted maximal HR (*y*-axis) was extrapolated as eVO_2max_. The relation “208 − 0.7 × age” [25] was applied to the age-predicted maximal HR for calculation. 

### 2.3. Data Analysis

Systematic differences between mVO_2max_ and eVO_2max_ were detected using Bland–Altman plots and linear regression analyses. Pearson correlation coefficients (*r*) were calculated to evaluate the relationship between mVO_2max_ and eVO_2max_. Error statistics, such as, constant error (*CE = ∑(Y –* $\hat{Y}$*)/N*), standard error of the estimate (*SEE* = *SD_Y_*$\sqrt{\left(1-r^{2}\right)}$), and total error (*TE* = $\sqrt{\sum {\left(Y-\hat{Y}\right)}^{2}/N}$), where *Y* is mVO_2max_ and $\hat{Y}$ is eVO_2max_, were compared among the estimation methods. All statistical analyses were performed using SAS version 9.4 (SAS Institute Japan, Tokyo, Japan) and Prism 9 (GraphPad Software, San Diego, CA, USA), with statistical significance set at a two-tailed *p*-value of <0.05.

## 3. Results

The participants’ average values of the anthropometric variables—mVO_2max_ when using the treadmill, the questionnaire’s PA score, and the JST’s HR index—are summarized separately for women and men (Table 1). The participants of both sexes generally had normal body sizes and CRF levels.

**Figure 1:**
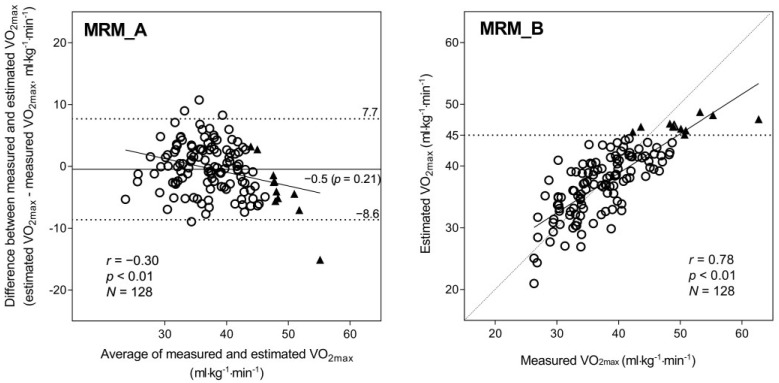
Bland–Altman plot (**A**) and correlation chart (**B**) with mVO_2max_ and eVO_2max_ using the MRM. In the Bland–Altman plots (**A**), the mean difference is shown as a solid line, and the 95% LoA is indicated as dashed lines; the regression line and correlation coefficient are also shown. In the correlation chart (**B**), the regression line and correlation coefficient are shown; the dashed line is the ideal line for the estimated and measured values. A total of 11 participants whose eVO_2max_ with the MRM exceeded 45.0 mL·kg^−1^·min^−1^ are shown with a black triangle in (**A**,**B**). Abbreviations: eVO_2max_, estimated VO_2max_; LoA, limit of agreement; MRM, multiple regression model; mVO_2max_, measured VO_2max_; and VO_2max_, maximal oxygen consumption.

**Figure 2:**
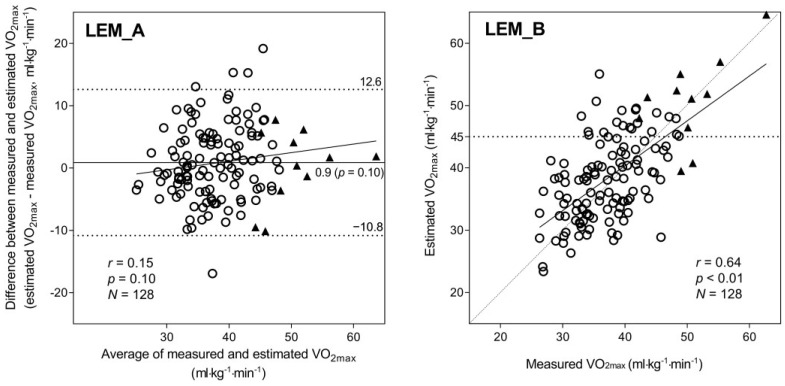
Bland–Altman plot (**A**) and correlation chart (**B**) with mVO_2max_ and eVO_2max_ using the LEM. In the Bland–Altman plots (**A**), the mean difference is shown as a solid line, and the 95% LoA is indicated as dashed lines; the regression line and correlation coefficient are also shown. In the correlation chart (**B**), the regression line and correlation coefficient are shown; the dashed line is the ideal line for the estimated and measured values. A total of 11 participants whose eVO_2max_ with the MRM exceeded 45.0 mL·kg^−1^·min^−1^ are shown with a black triangle in (**A**,**B**). Abbreviations: eVO_2max_, estimated VO_2max_; LEM, linear extrapolation method; LoA, limit of agreement; MRM, multiple regression model; mVO_2max_, measured VO_2max_; VO_2max_, maximal oxygen consumption.

The Bland–Altman plots revealed systematic differences between mVO_2max_ and eVO_2max_. The MRM (Figure 1A) exhibited a non-significant fixed bias (−0.47 mL·kg^−1^·min^−1^, *p* = 0.21) and a significant proportional bias (*r* = −0.30, *p* < 0.01), which increased at higher VO_2max_ values. The LEM (Figure 2A) also showed both fixed bias (0.89 mL·kg^−1^·min^−1^, *p* = 0.10) and proportional bias (*r* = 0.15, *p* = 0.10); however, neither was significant. The range of 95% limits of agreement (LoA) for the LEM was clearly wider than that for the MRM, suggesting a lower estimation accuracy. Indeed, the regression analysis showed a strong correlation between mVO_2max_ and eVO_2max_ values using both the MRM (*r* = 0.78, Figure 1B) and LEM (*r* = 0.64, Figure 2B); nonetheless, the correlation was stronger when the MRM was used. As shown in Figure 1B, the MRM also produced eVO_2max_ values under the ideal line (*Y* = *X*) for 9 out of 11 participants with the highest fitness (VO_2max_ ≥ 45.0 mL·kg^−1^·min^−1^, indicated by black triangles in all figures). On the other hand, several values derived using the LEM were near the ideal line for the same 11 participants (Figure 2B). Thus, the MRM tended to underestimate the VO_2max_ in fit individuals, whereas the LEM showed no proportional bias but generally yielded less accurate VO_2max_ estimates.

**Figure 3:**
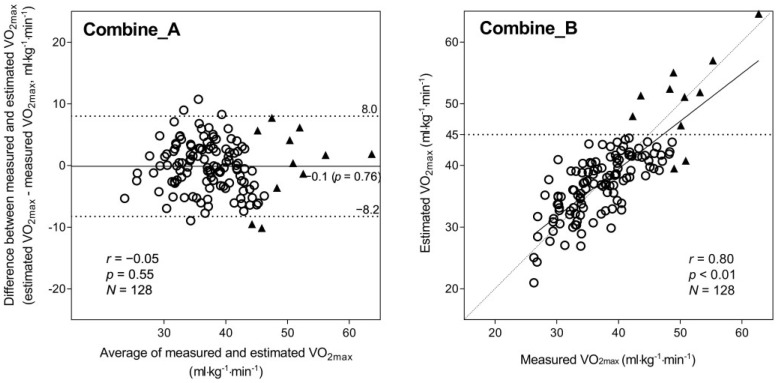
Bland–Altman plot (**A**) and correlation chart (**B**) with mVO_2max_ and eVO_2max_ using the combined method. In the Bland–Altman plots (**A**), the mean difference is shown as a solid line, and the 95% LoA is indicated as dashed lines; the regression line and correlation coefficient are also shown. In the correlation chart (**B**), the regression line and correlation coefficient are shown; the dashed line is the ideal line for the estimated and measured values. A total of 11 participants whose eVO_2max_ with the MRM exceeded 45.0 mL·kg^−1^·min^−1^ are shown with a black triangle in (**A**,**B**). Abbreviations: eVO_2max_, estimated VO_2max_; LoA, limit of agreement; MRM, multiple regression model; mVO_2max_, measured VO_2max_; and VO_2max_, maximal oxygen consumption.

We speculated that the most accurate eVO_2max_ could be obtained through the use of a combined model in which the LEM corrected for VO_2max_ underestimation by the MRM in participants with high fitness. The procedure was as follows: (1) the eVO_2max_ for each participant was calculated using the MRM, and (2) if the eVO_2max_ exceeded 45.0 mL·kg^−1^·min^−1^, then it was recalculated using the LEM. Figure 3 shows the Bland–Altman plot (A) and scatter plot (B) constructed using this procedure. With this combined method, neither the fixed bias (−0.11 mL·kg^−1^·min^−1^, *p* = 0.76) nor the proportional bias (*r* = −0.05, *p* = 0.55) were significant, and the correlation between mVO_2max_ and eVO_2max_ was still strong (*r* = 0.80).

Table 2 compares the other error statistics (CE, SEE, and TE) among the three estimation methods. The CE was not significant for all three methods; however, a larger difference between mVO_2max_ and eVO_2max_ was observed with the LEM than with the other two methods. The SEE increased in the order of LEM, MRM, and combined method. This tendency was also observed for the TE. Additionally, similar values were obtained between the SEE and TE for the MRM and combined method, whereas the TE was relatively higher than the SEE in the LEM.

## 4. Discussion

The high correlation coefficient (*r* = 0.78) between the MRM eVO_2max_ and mVO_2max_ indicated that the equation derived from 173 adults in a previous study [16] could be applied to an independent participant group (i.e., good stability of the equation). The MRM yielded greater estimation accuracy; however, a substantial proportional bias was also detected, resulting in a remarkable VO_2max_ underestimation in participants with high fitness (Figure 1). On the other hand, the LEM demonstrated lower estimation accuracy than the MRM but no proportional bias—that is, the LEM did not underestimate VO_2max_ in participants with high fitness (Figure 2). Interestingly, the combined method (Figure 3), which replaces the MRM with the LEM for predicted values ≥ 45 mL·kg^−1^·min^−1^ yields an optimal result, suggesting that the inaccuracies induced by the MRM and LEM could be partly mitigated by optimizing the statistical model. Thus, VO_2max_ could be predicted with a relatively high degree of accuracy from a simple combination of morphometric measurements, questionnaire data, and step-test HR by combining MRM and LEM calculations. 

One metabolic equivalent (MET) change in CRF is a meaningful value for disease prevention [26]. Hence, it would be a reasonable policy that the target SEE of VO_2max_ estimation models should be ≤1.0 MET (3.5 mL·kg^−1^·min^−1^). Peterman et al. [11] reported SEEs ranging from 4.1 to 6.2 mL·kg^−1^ min^−1^ for non-exercise models, indicating substantial variation in the validity of these questionnaires for VO_2max_ estimation. Some studies [7,8] suggested that eVO_2max_ questionnaires should include items assessing the frequency, duration, and intensity of PA and that, owing to its greater influence on eVO_2max_, the intensity score should be more heavily weighted than the frequency and duration scores. One of the previous studies [7] that used the same questionnaire as the present study reported an SEE of 4.29 mL·kg^−1^·min^−1^ for the non-exercise MRM (including age, sex, BMI, and the questionnaire’s PA score), as well as excellent test–retest reliability (intraclass correlation coefficient [ICC], 0.87; 95% confidence interval [CI], 0.82–0.91) for the PA score. Meanwhile, several studies have shown that evaluating “changes” in VO_2max_ is difficult to perform using a questionnaire-based, non-exercise estimation method. For instance, Peterman et al. [27] found limited accuracy for 27 non-exercise prediction equations as compared with that for directly measured values in a cohort of 987 healthy adults. Similarly, Lannoy and Ross [10] reported limited VO_2peak_ estimation accuracy, as compared with that for mVO_2peak_, in 163 adults participating in a 24 week exercise intervention. These studies suggest that biological measures such as the HR may be required for accurate VO_2max_ estimation.

The HR during the step test is often used for VO_2max_ estimation. The increase in HR during a stepping exercise will be lower and will more rapidly return to baseline in individuals with high fitness than in those with low fitness [14]. The Queen’s College step test [28], the Astrand–Ryhming step test [29], and the Chester step test [17] assume that the HR during or soon after exercise is lower in individuals with high fitness. On the other hand, others, such as the Harvard step test [30] and YMCA step test [31], assume that the HR decreases more rapidly during recovery in individuals with high fitness. However, the use of recovery HR alone may not be sufficiently sensitive for precisely predicting VO_2max_ [14,32]. The JST’s HR index in the present study captures the HR responses for VO_2max_ estimation both during the stepping exercise and during the recovery period. A recent study [14] reported that the SEE for the JST (4.54 mL·kg^−1^·min^−1^) was relatively lower than the SEE for the Chester step test (4.99 mL·kg^−1^·min^−1^) and that the JST’s HR index demonstrated fair-to-good test–retest reliability (ICC, 0.65; 95% CI, 0.53–0.74). 

Recently, Webb et al. [15] reported that the addition of both the questionnaire score and step-test HR to the MRM (including sex and body weight) improved the accuracy of eVO_2max_. Similarly, Matsuo et al. [16] showed that the addition of the JST’s HR index to a questionnaire-based, non-exercise MRM (including age, sex, BMI, and the questionnaire’s PA score) improved the accuracy of eVO_2max_. Nevertheless, the study [16] suggested that this “synergistic effect” of the questionnaire’s PA score and step-test HR for MRM eVO_2max_ was not effective in detecting changes in mVO_2max_ in an intervention experiment. That is, the study showed that mVO_2max_, which was measured using a cycling ergometer and gas analyzer, increased by approximately 20% during the exercise training period and decreased by approximately 10% during the subsequent detraining period. The MRM eVO_2max_ apparently underestimated the increase in mVO_2max_ owing to a substantial proportional bias; therefore, the MRM could not detect changes in the CRF along with lifestyle modifications. Additionally, the study also showed that the LEM eVO_2max_ presumably detected the increase in mVO_2max_ while yielding a larger error variance than the MRM.

The results of the present study are consistent with the findings of a previous study [16]. The MRM exhibited good VO_2max_ estimation accuracy in participants with average or low fitness but significantly underestimated the VO_2max_ in participants with high fitness (Figure 1). On the other hand, with the LEM (Figure 2), the eVO_2max_ values of participants with high fitness were distributed near the ideal line (i.e., no underestimation); however, the LEM yielded larger errors across the VO_2max_ range, likely because of greater errors in the age-predicted maximal HR [21,22], which is heavily weighted in the LEM calculation process. Therefore, in the present study, we propose a method combining the advantages of both the MRM and LEM—that is, the generally higher accuracy of the MRM when eVO_2max_ is below a certain cut-off (in this case, 45 mL·kg^−1^·min^−1^) and the absence of proportional bias in the LEM estimates when VO_2max_ is above the cut-off. Consequently, the significant proportional bias observed for the MRM (Figure 1A) disappeared, and the wider LoA seen for the LEM (Figure 2A) was improved by the combined method (Figure 3A). Additionally, the combined method produced an improved scatter plot as well as the highest correlation coefficient among the three methods. Based on the principles of cross-validation analyses [33,34], the SEE and TE values should be calculated because the TE reflects the actual difference between measured and estimated values, whereas the SEE reflects only the variation in regression; similar values between the SEE and TE reflect a close approximation between the regression line and the line of identity. From this viewpoint, we compared the error statistics of the three methods (Table 2). Consequently, better CE, SEE, and TE values were observed for the combined method, and the difference between the SEE and TE for the combined method was not large. Thus, the favorable error statistic values demonstrate the superiority of the combined method.

This study has some limitations. First, the participants decided to participate after viewing our research advertisement, which might have likely introduced a selection bias in adults seeking to monitor their own CRF value. Second, mVO_2max_ was measured only once in each participant; hence, there was no estimation of intra-individual variability or possible measurement errors. Third, this study primarily included healthy participants and did not include any individuals having medical conditions precluding VO_2max_ testing or using medical drugs, such as β-blockers. Therefore, the estimation method proposed in this study might not apply to those patients and should be further investigated. Fourth, it should be considered that the favorable results obtained by the combined method may have happened by chance because of the unique dataset used in the present study. Similarly, the cut-point (≥45 mL·kg^−1^·min^−1^) may not be applicable to other populations. Therefore, the combined method should be validated using different subjects’ data. Even so, we believe that the combined method presented in this study may be a feasible method for improving the accuracy of VO_2max_ estimation.

## 5. Conclusions

The present study showed that the MRM yielded higher estimation accuracy than the LEM but demonstrated marked proportional bias (i.e., VO_2max_ underestimation in participants with high fitness). However, this weakness could be mitigated through the use of the LEM within a limited range because the proportional bias was less likely to occur in the LEM. Further research is needed to confirm that this approach is applicable to other populations.

## Figures and Tables

**Table 1:** Characteristics of the study participants.

	Females (*N* = 60)			Males (*N* = 68)			Total (*N* = 128)		
Age, years	48.3	±	7.0	48.3	±	6.9	48.3	±	6.9
Height, cm	158.6	±	5.8	170.7	±	5.1	165.0	±	8.1
Body weight, kg	54.4	±	9.1	71.7	±	9.7	63.6	±	12.8
BMI, kg·m^−2^	21.6	±	3.2	24.6	±	2.9	23.2	±	3.4
VO_2max_, mL·kg^−1^·min^−1^	34.4	±	5.0	41.2	±	6.3	38.0	±	6.6
VO_2max_, L·min^−1^	1.86	±	0.30	2.93	±	0.44	2.42	±	0.66
Questionnaire’s PA score, points	8.6	±	7.5	11.9	±	7.9	10.3	±	7.9
HR index, step test score, bpm	46.9	±	15.0	35.4	±	9.3	40.8	±	13.6

Values are presented as mean ± standard deviation. BMI, body mass index; HR, heart rate; PA, physical activity; and VO_2max_, maximal oxygen consumption.

**Table 2:** Measured versus estimated VO_2max_ and error statistics (*N* = 128).

Measured VO_2max_ (mL·kg^−1^·min^−1^)				Estimated VO_2max_ (mL·kg^−1^·min^−1^)			CE (*p*-Value)				SEE	TE
38.0	±	6.6	MRM	37.6	±	5.4	0.5	±	4.2	(0.21)	4.15	4.17
			LEM	38.9	±	7.4	−0.9	±	6.0	(0.10)	5.08	6.02
			Combined	37.9	±	6.4	0.1	±	4.1	(0.76)	3.99	4.13

Values are presented as mean ± standard deviation. CE, constant error; SEE, standard error of the estimate; TE, total error; and VO_2max_, maximal oxygen consumption.

## Data Availability

The derived data supporting the findings of this study are available from the corresponding author upon reasonable request following the acquisition of approval from the Ethical Committee of the National Institute of Occupational Safety and Health, Japan.

## Supplementary Materials

The following supporting information can be downloaded at: https://www.mdpi.com/article/10.3390/jcdd10010009/s1, Table S1: Validated questionnaire for VO_2max_ estimation.
