# Supplementary material for: Improved VO2max Estimation by Combining a Multiple Regression Model and Linear Extrapolation Method
Source: J Cardiovasc Dev Dis. 2022 Dec 27;10(1):9. doi: 10.3390/jcdd10010009 (PMC9865627; doi:10.3390/jcdd10010009)
Supplement: Supplementary file 1 [file jcdd-10-00009-s001.zip › jcdd-2065812-supplementary.pdf]

Journal of
Cardiovascular Development and Disease

MDPI

Supplementary Materials

Improved VO_{2max} Estimation by Combining a Multiple Regression Model and Linear Extrapolation Method

Tomoaki Matsuo, Rina So and Fumiko Murai

Table S1. Validated questionnaire for VO_{2max} estimation.

	Questions	Answer options (points*)
Q1	How frequently on average do you engage in heart rate-increasing physical tasks during working hours?	1. never/almost never (0)
		2. rarely (3)
		3. sometimes (5)
		4. often (10)
Q2	In your leisure time on a workday,how much intentional physical activity do you engage in?	1. never/almost never (0)
		2. 1–3 days a month (1)
		3. 1 or 2 days a week (2)
		4. ≥ 3 days a week (3)
Q3	If options 2–4 were selected from Q2,please provide the average exercise time per day	1. < 15 min (1)
		2. 15–30 min (2)
		3. 31–60 min (3)
		4. > 60 min (4)
		N/A (Q2 =1)
Q4	If options 2–4 were selected from Q2,please tell us the approximate intensity of the exercise per session	1. no sweating or panting (0)
		2. sweating and panting (3)
		3. strained breathing (5)
		4. to the point of exhaustion(10)
		N/A (Q2 =1)
Q5	On holidays, how much intentional physical activity do you engage in?	1. never/almost never (0)
		2. 1–2 days a month (1)
		3. once a week (2)
		4. ≥2 days a week (3)
Q6	If options 2–4 were selected from Q5,please provide the average exercise time per day	1. < 15 min (1)
		2. 15–30 mins (2)
		3. 31–60 mi (3)
		4. > 60 min (4)
		N/A (Q5 =1)
Q7	If options 2–4 were selected from Q5,please tell us the approximate intensity of the exercise per session	1. no sweating or panting (0)
		2. sweating and panting (3)
		3. strained breathing (5)
		4. to the point of exhaustion(10)
		N/A (Q5 =1)

*If the respondent selects option #1 on Q2, the scores on Q3 and Q4 are zero. Similarly, if the respondent selects option #1 on Q5, the scores on Q6 and Q7 are zero. The PA score (0–44 points) is calculated by adding the points from Q1–Q7.
